# Human Detection Based on the Generation of a Background Image by Using a Far-Infrared Light Camera

**DOI:** 10.3390/s150306763

**Published:** 2015-03-19

**Authors:** Eun Som Jeon, Jong-Suk Choi, Ji Hoon Lee, Kwang Yong Shin, Yeong Gon Kim, Toan Thanh Le, Kang Ryoung Park

**Affiliations:** Division of Electronics and Electrical Engineering, Dongguk University, 26 Pil-dong 3-ga, Jung-gu, Seoul 100-715, Korea; E-Mails: jeunsom@dgu.edu (E.S.J.); jjongssuk@dgu.edu (J.-S.C.); easygns@dgu.edu (J.H.L.); skyandla@dongguk.edu (K.Y.S.); csokyg@dongguk.edu (Y.G.K.); lethanhtoan@dgu.edu (T.T.L.)

**Keywords:** human detection, thermal camera image, generation of background image, background subtraction, adaptive threshold

## Abstract

The need for computer vision-based human detection has increased in fields, such as security, intelligent surveillance and monitoring systems. However, performance enhancement of human detection based on visible light cameras is limited, because of factors, such as nonuniform illumination, shadows and low external light in the evening and night. Consequently, human detection based on thermal (far-infrared light) cameras has been considered as an alternative. However, its performance is influenced by the factors, such as low image resolution, low contrast and the large noises of thermal images. It is also affected by the high temperature of backgrounds during the day. To solve these problems, we propose a new method for detecting human areas in thermal camera images. Compared to previous works, the proposed research is novel in the following four aspects. One background image is generated by median and average filtering. Additional filtering procedures based on maximum gray level, size filtering and region erasing are applied to remove the human areas from the background image. Secondly, candidate human regions in the input image are located by combining the pixel and edge difference images between the input and background images. The thresholds for the difference images are adaptively determined based on the brightness of the generated background image. Noise components are removed by component labeling, a morphological operation and size filtering. Third, detected areas that may have more than two human regions are merged or separated based on the information in the horizontal and vertical histograms of the detected area. This procedure is adaptively operated based on the brightness of the generated background image. Fourth, a further procedure for the separation and removal of the candidate human regions is performed based on the size and ratio of the height to width information of the candidate regions considering the camera viewing direction and perspective projection. Experimental results with two types of databases confirm that the proposed method outperforms other methods.

## 1. Introduction

The need for computer vision-based human detection has increased in fields, such as security, intelligent surveillance and monitoring systems [1,2,3,4,5]. However, performance enhancement of human detection based on visible light cameras is limited because of factors, such as nonuniform illumination, shadows and low external light in the evening and night. Consequently, thermal cameras have been widely used for tasks, such as security and outdoor surveillance. In general, the infra-red (IR) spectrum can be classified into four sub-bands, such as near-IR (NIR), whose wavelength ranges from 0.75 to 1.4 μm, short-wave IR (SWIR), whose wavelength ranges from 1.4 to 3 μm, medium-wave IR (MWIR), whose wavelength ranges from 3 to 8 μm, and long-wave IR (LWIR), whose wavelength ranges from 8 to 15 μm [6]. Because significant heat energy is reported to be emitted in MWIR and LWIR sub-bands, both of these sub-bands are usually used for sensing the human body, including the face, without an additional light source, and they are referred to as the thermal sub-band [6]. We call the image acquired in this sub-band the thermal image in this paper.

The gray level of an object in a thermal image depends on the temperature of the object. If the object is hotter than the surrounding environment, it is represented as a brighter region. Typically, the gray level of human areas in a thermal image appears differently from the background, allowing thermal images to be applied to the detection of human areas in an image. However, under different conditions, the characteristics of the human areas and background can change. This can influence the accuracy of detecting the precise locations and shapes of the human areas in the thermal image [7,8,9,10,11,12,13,14,15].

Recently, significant work has focused on detecting human areas in thermal camera images, because the performance of the detection influences the accuracy of the human tracking and behavioral recognition. Previous research into human detection in thermal camera images can be classified into two categories: without background generation [2,15,16,17,18,19,20] and with background generation [21,22,23,24,25,26,27,28,29]. For the former category, Martin *et al.* proposed a detector and tracker of objects based on motion and scale-invariant feature transform (SIFT) methods [2]. In addition, the former used the methods for feature extraction based on the histogram of the oriented gradient (HOG) [7,8,9,15,16,17] and geometric characteristics [15] with a classifier based on support vector machine (SVM) [7,15,17,18], the adaptive boosting (Adaboost) method [19] and the soft-label boosting algorithm [20]. The advantage of these methods is that they can detect an object without a background image. However, they have the disadvantage that they require a pre-defined template or classifier for human areas; this must be obtained through training. Moreover, their performance is influenced by environmental factors, such as rain, snow and the amount of sunlight, and they require an excessive processing time to detect the human areas in the entire image by scanning.

Therefore, techniques for detecting human areas using background generation can be employed as an alternative. These are based on a Gaussian background-subtraction approach [21,22,24,25], texture change [23], expectation minimization (EM) [26,27] and image averaging [28].

To detect an object, methods, such as a contour saliency map (CSM) [21,24,25], CSM template matching [22], shape and appearance-based detection [26,27], spatiotemporal texture vectors [23] and a boosting framework [28], can be applied. In some research [26,27], shape descriptors, such as compactness and leanness, are obtained from the skeleton of the object, and they are used as the input values of SVM for shape-based detection. In addition, the features obtained by principal component analysis (PCA) with the pixel values from a window of fixed size [26] and varying size [27] are used for appearance-based detection. Using both shape and appearance-based detection is one of the main novelties of their research. In previous research [29], a particle filter framework and histogram based on the intensity-distance projection space were used. The advantage of these methods is that they are applicable to multiple environmental conditions and can detect objects of varying scale. However, the disadvantage is that performance degradation can occur if the intensity of the object is similar to the background. Further, if there exist motionless humans (at the same positions) in all of the image frames, these approaches can produce an erroneous background, which has the areas of the image occupied by motionless humans, although the accurate background should not include the areas of the image occupied by humans. Moreover, they do not consider the parameters of the detector, which are adaptively determined by the background information. The pixel brightness of the background is usually affected by environmental temperature. That is, the pixel brightness of the background is higher in the daytime than that that at dawn or night. If the pixel brightness is too high or too low, the difference of the pixel brightness between the background and human usually decreases. Therefore, the thresholds used for obtaining the binarization image from background subtraction should be reduced in these cases. This scheme means that the parameters of the detector of the human by background subtraction are adaptively determined by the background information (the pixel brightness of the background). Other research proposed the usage of stereo thermal cameras [10] and a dual system of visible light and thermal cameras [11]. However, these systems are expensive and oversized, owing to the use of two cameras.

**Table 1:** Comparison of previous and the proposed methods. HOG, histogram of the oriented gradient; EM, expectation minimization; CSM, contour saliency map.

Category	Without Background Generation [7,8,9,15,16,17,18,19,20]	With Background Generation	
		Not Adjusting the Parameters for Detection Based on Background Information [21,22,23,24,25,26,27,28,29]	Adjusting the Parameters for Detection Based on Background Information (Proposed Method)
Examples	- Motion + SIFT-based [2], HOG-based [7,8,9,15,16,17], geometric characteristics-based [15,17,18], Adaboost-based [19] and soft-label boosting-based [20] detection of object.	- Gaussian model-based [21,22,23,25], texture change-based [23], EM-based [26,27] and image averaging-based [28] background modeling and subtraction.	- The correct background image can be generated by image averaging, various filtering and erasing of the human area with adaptive determination of thresholds and parameters for the human detector.
		- CSM-based [21,24,25], CSM template matching-based [22], shape and appearance-based [26,27], spatiotemporal texture vectors-based [23], boosting framework-based [28] and particle filter and histogram-based [29] detection of object.	
Advantages	- Can detect object without a background image.	- Can be applicable to various environmental conditions and can detect objects of various scales.	- Robust detection of the human area can be obtained by adaptively determining the thresholds and parameters for detection considering background information.
			- Does not require a training procedure to obtain the classifier of human detection.
Disadvantages	- Requires a pre-defined template or classifier for the human area that must be obtained through training.	- Performance degradation can occur if the intensity of the object is similar to the background.	- Additional procedure is required for obtaining correct background image.
	- Performance is influenced by various environmental factors, such as rain, snow and the amount of sunlight.	- The approach can produce erroneous background, including the image areas occupied by humans, if the image frames include the image areas occupied by motionless humans.	
	- Requires significant processing time to detect the human area in the entire image by scanning.	- Parameters for the detector of the image areas occupied by humans are not adaptively determined based on background information.	

To address these problems, we present a new method of detecting human areas in a thermal image in varying environmental conditions. The proposed approach is based on the method of background subtraction. One background image is generated using median and average filtering. Additional filtering procedures based on maximum gray level, size filtering and region erasing are applied to remove human areas from the background image. Candidate human regions in the input image are located by combining the pixel and edge difference images between the input and background images. The thresholds for the difference images are adaptively determined based on the brightness of the generated background image. Noise components are removed by component labeling, a morphological operation and size filtering. The contiguous foreground area may be occupied by more than a single human. In addition, the size of this area can be too small to be accepted as the foreground area, which is caused by the incorrect detection of the foreground area. Therefore, these detected areas are merged or separated by the further procedure based on the information in the horizontal and vertical histograms of the detected area. This procedure is adaptively operated based on the brightness of the generated background image. Finally, a further procedure for the separation and removal of the candidate human regions is performed based on the information of the size and the ratio of the height to width of the candidate regions considering the camera viewing direction and perspective projection. 

Table 1 presents a summarized comparison of the previous research of human detection in thermal camera images and the proposed method.

This paper is organized as follows. We provide an overview of the proposed algorithm in Section 2. We present experimental results and analysis in Section 3. Finally, conclusions are presented in Section 4.

## 2. Proposed Method

### 2.1. Proposed Method

**Figure 1:**
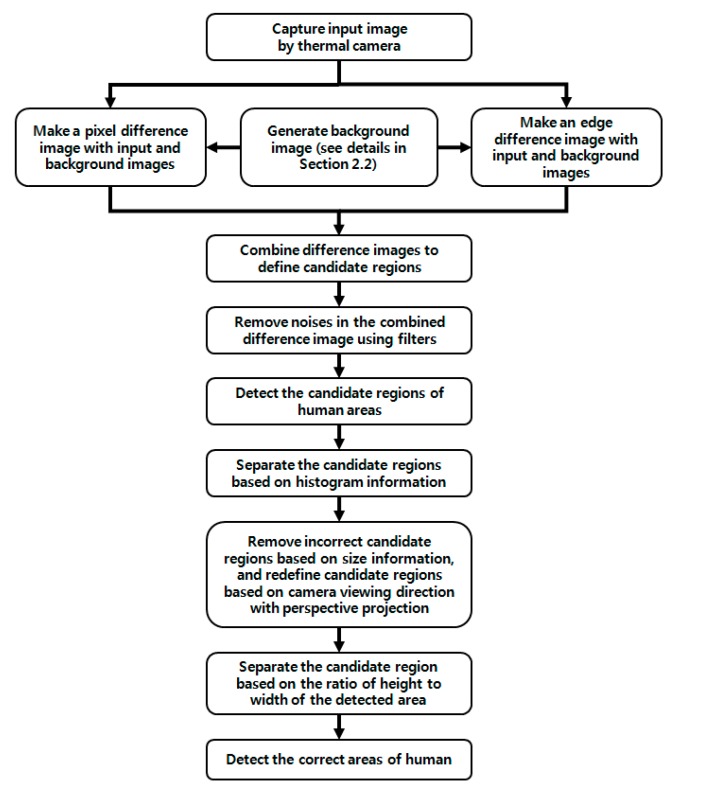
Overall procedure of the proposed method.

An overview of the proposed method is presented in Figure 1.

Our approach for detecting humans in a thermal image can be divided into three steps: generating a background image, obtaining a difference image with the background and input image and detecting humans in the difference image. As explained in Section 1, we call the image acquired in the sub-bands of MWIR and LWIR the thermal image [6] in this paper.

To begin, a background image is generated. In this step, a correct background image is obtained by various filtering and erasing of human areas (see the details in Section 2.2). Then, two (pixel and edge) difference images are obtained from the background and input images. These two binary difference images are combined using pixel-wise conjunction. The thresholds for the difference images are adaptively determined based on the brightness information of the generated background image (see the details in Section 2.3).

The third step addresses human detection in the combined difference image. After applying size filtering and a morphology operation based on the size of the candidate areas, noise is removed. The remaining areas are separated by a vertical and horizontal histogram of the detected regions using the intensity of the background. Detected regions that may have more than two human areas are merged. Therefore, further procedures are performed to separate the candidate regions and to remove noise regions. These are based on the information of the size and ratio (of the height to width) of the candidate regions considering the camera-viewing angle and perspective projection (see the details in Section 2.4). Finally, we obtain the correct human areas.

### 2.2. Generating a Background Image

Research into detecting humans has focused on creating background models to detect candidate regions of human areas [21,22,23,24,25,26,27,28,29]. Generating a robust background image is essential to extract candidate regions of human areas based on background subtraction. However, the methods of creating a background image from a sequence containing motionless humans in all frames have a problem in that an incorrect background is generated with a human included. This influences the performance of detecting a human based on the background-subtraction method. To solve this problem, two images from different sequences are averaged to obtain a background image [26]. This, however, has the problem that the brightness of the generated background image is changed compared to the input image, causing a human area detection error. For example, if the first image is from the sequence captured in the daytime and the second one is from the sequence captured at night, the pixel values, even in the same position, are different from each other in these two images. That is because the pixel value in the thermal image is usually higher in the daytime than that at night. Therefore, if a temporal-averaged image is obtained from these two images as a background image, the pixel value in each position of the background image is different from that of the first or second image. Therefore, although a current input image is obtained in the daytime or at night, the pixel value in each position of the current input image is different from that of the background image, even at the same position without humans. Consequently, these pixel differences produce a lot of erroneous regions as the human area by background subtraction.

**Figure 2:**
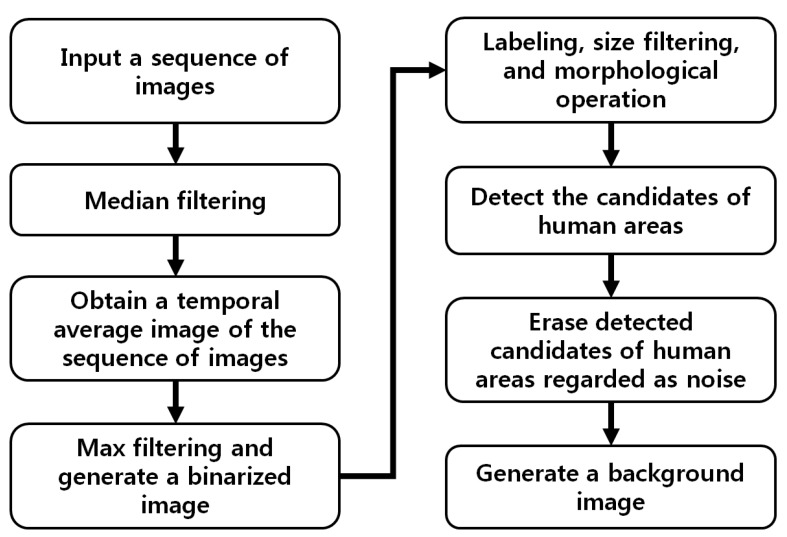
Flow chart of generating a background image.

Therefore, we propose an approach for generating a correct background image under these conditions. A flow chart of the proposed method is presented in Figure 2.

**Figure 3:**
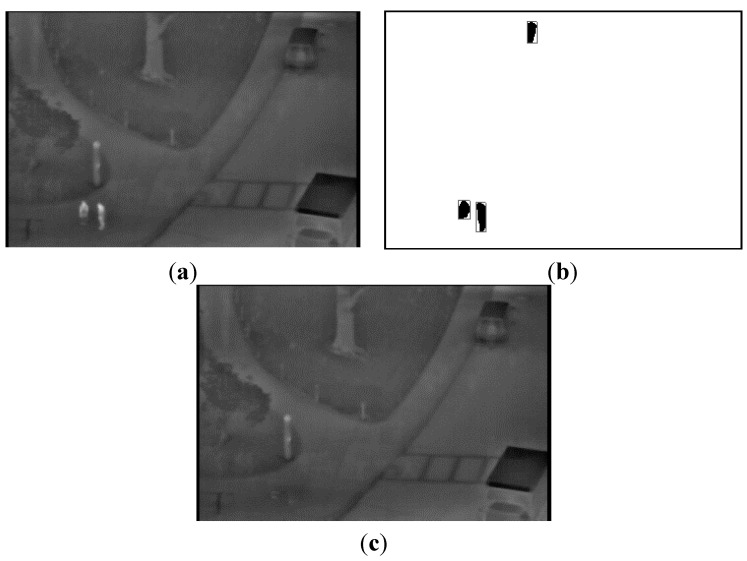
Examples of obtaining the background image from an open database: (**a**) preliminary background image obtained by temporal averaging; (**b**) extracted human areas by the binarization, labeling, size filtering and a morphological operation of Figure 2; and (**c**) the generated final background image.

In the proposed research, one background image is generated with training before testing. In detail, one background image is generated using training images, whereas the performance of our method is measured using the testing images that are not used for generating the background image. Although the training images include both background and humans, an accurate background image (excluding the image areas occupied by humans) is obtained by our method in Figure 2. The sequence of training images is processed by a median filter of 3 × 3 pixels to reduce noise; pixels with the same positions as the training images are temporally averaged as illustrated in Figure 3a. In previous research [28], Calafut *et al.* obtained the background image using the simple operation of temporal averaging. However, an incorrect background is generated when areas of motionless humans are included in the image sequence. Therefore, we propose a method for obtaining the correct background image by including an additional procedure to remove human areas in the background image as follows. Normally, a human area has the characteristics that its gray level is higher than the background area in thermal image. In detail, the pixel of higher temperature usually has a larger value than that of lower temperature in the thermal image. Therefore, because humans actively emit heat, the corresponding pixel values usually have a larger one in the thermal image compared to those of the background.

Based on this, to force the human area to be more distinctive compared to the background region, we apply a 3 × 3 local max filter (that selects a pixel if it has the maximum value in the window) to the background image. Then, the processed image is converted to a binarized version based on the threshold method of Equation (3) [30]:

(1) $${\text{μ}}_{k}=\;\frac{\sum_{i=1}^{M}\sum_{j=1}^{N}I_{k}\left(i,j\right)}{M\times N}$$

(2) $${\text{σ}}_{k}=\;\sqrt{\frac{\sum_{i=1}^{M}\sum_{j=1}^{N}{\left(I_{k}\left(i,j\right)-{\text{μ}}_{k}\right)}^{2}}{M\times N-1}}$$

(3) $$B_{k}\left(i,j\right)=\{\;\begin{array}{c} 0\;if\;I_{k}\left(i,j\right)>{\text{μ}}_{k}+Th\cdot {\text{σ}}_{k}\\ 1\;otherwise \end{array}$$

where *I_k_*(*i*, *j*) is the gray value of the pixel at the position (*i*, *j*). *M* and *N* are the width and height of the image, respectively. μ*_k_* and σ*_k_* are the average value and standard deviation of the image pixel, respectively. *B_k_*(*i*, *j*) is a binary image, and *k* is the number of the input image in the sequence. *Th* is the optimal parameter, which is experimentally determined.

Then, the candidates of human areas are detected as shown in Figure 3b. As shown in Figure 3b, most of the detected candidate areas represent the humans, and these areas should not be included in the generated background image. Therefore, our method removes them by the procedure of erasing the areas. A detailed explanation of the erasing algorithm follows. By horizontal scanning, the left- and right-most positions of each candidate area per each row are located. Then, the pixels whose X positions are smaller and larger than the left- and right-most positions are determined as the pixels of the nonhuman region, respectively. Finally, the candidate area per each row is erased by linear interpolation with these pixels. This procedure is iterated within the entire image.

For example, we assume that the left-upper-most position of the image is (0, 0) at the (*x*, *y*) coordinate, and one candidate area is composed of seven pixels, such as (50, 1), (51, 1), (52, 1), (49, 2), (50, 2), (51, 2) and (52, 2), respectively. Then, there is no detected left- and right-most position by the horizontal scanning of the 1st row. By the horizontal scanning of the 2nd row, the left- and right-most positions are detected as (50, 1) and (52, 1), respectively. Then, (48, 1) (the pixel whose X position is smaller than the left-most position) and (54, 1) (the pixel whose X position is larger than the right-most position) can be determined as the pixels of the nonhuman region, respectively. If the gray levels at the positions (48, 1) and (54, 1) are 10 and 70, respectively, the candidate area of the 2nd row is erased by linear interpolation with 10 and 70. That is, the gray values of 10, 20, 30, 40, 50, 60 and 70 are newly assigned to the pixels of (48, 1), (49, 1), (50, 1), (51, 1), (52, 1), (53, 1) and (54, 1), respectively. This procedure is iterated from the 3rd row to the last row. Through this procedure, the discontinuous boundary between the human candidate area and its neighboring background can disappear, and the consequent candidate area can be removed.

From these methods, a correct background image is generated, even though motionless humans exist in all of the images at the same location. In the proposed research, we used two kinds of databases, an open database and our own database. Detailed descriptions of these databases are provided in Section 3.1. An example of the final background image with the open database is displayed in Figure 3c. We can observe that the background image does not include the human areas.

**Figure 4:**
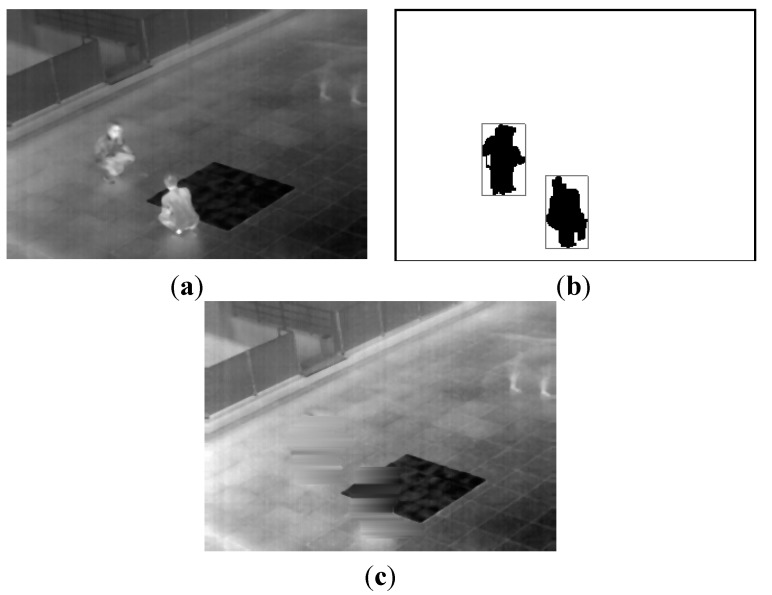
The first example for obtaining a background image from our database: (**a**) preliminary background image obtained by temporal averaging; (**b**) extracted human areas; and (**c**) the generated final background image.

**Figure 5:**
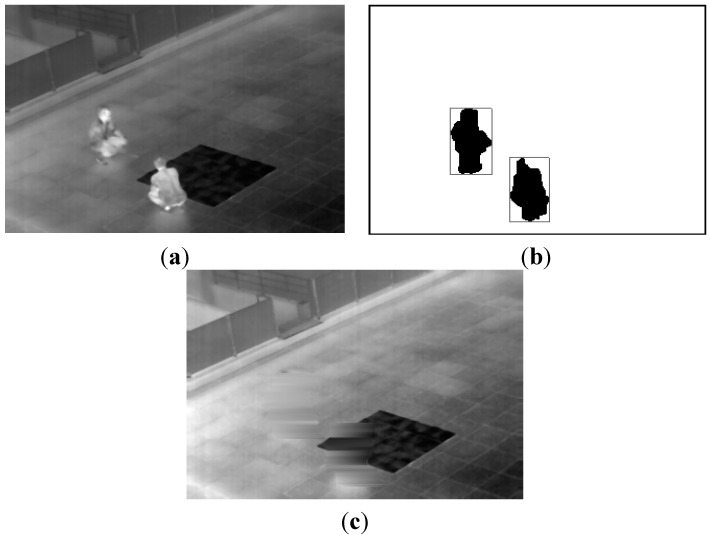
The second example for obtaining a background image from our database: (**a**) preliminary background image obtained by temporal averaging; (**b**) extracted human areas; and (**c**) the generated final background image.

In Figure 4 and Figure 5, we present additional examples for obtaining the background images using the proposed method with our database. We can confirm that the proposed method can generate correct backgrounds that do not include human areas.

### 2.3. Generating a Difference Image with the Background and Input Image

To identify the regions-of-interest (ROI) that contain humans, we use a background-subtraction technique, as illustrated in Figure 1. First, we apply median and average filters of 3 × 3 pixels to an input image to reduce noise. A pixel difference image is created with the pixels between the background and input images using optimal thresholds based on the intensity of the background image. The operator is presented in Equations (4) and (5).

(4) $$\overset{M}{\underset{i=1}{\sum }}\overset{N}{\underset{j=1}{\sum }}B_{k}(i,j)>T\;and\;\overset{M}{\underset{i=1}{\sum }}\overset{N}{\underset{j=1}{\sum }}\{I_{k}\left(i,j\right)-B_{k}(i,j)\}>U$$

where *B_k_*(*i*, *j*) and *I_k_*(*i*, *j*) are the pixel gray levels of a generated background and an input image at the position (*i*, *j*), respectively. *M* and *N* are the width and height of the image, respectively. *k* is number of the input image in the sequence. *T* and *U* are the optimal thresholds, which were experimentally determined. If Equation (4) with pixel *I_k_*(*i*, *j*) is satisfied, a binarized image (*D_k_*(*i*, *j*)) is obtained using Equation (5).

(5) $$D_{k}\left(i,j\right)=\{\begin{array}{c} \;1\;if\;\left|I_{k}\left(i,j\right)-B_{k}\left(i,j\right)\right|>X\\ 0\;otherwise\; \end{array}$$

where *X* is an optimal threshold that is adaptively determined based on the brightness of the background image. In detail, with too large or small of a value of the brightness of the background image, the proposed system assigns a smaller value to *X*, because the pixel difference between the input and background image usually decreases in these cases. This adaptive scheme enables the detection of a human area by background subtraction to be robust to various environmental conditions of the background.

**Figure 6:**
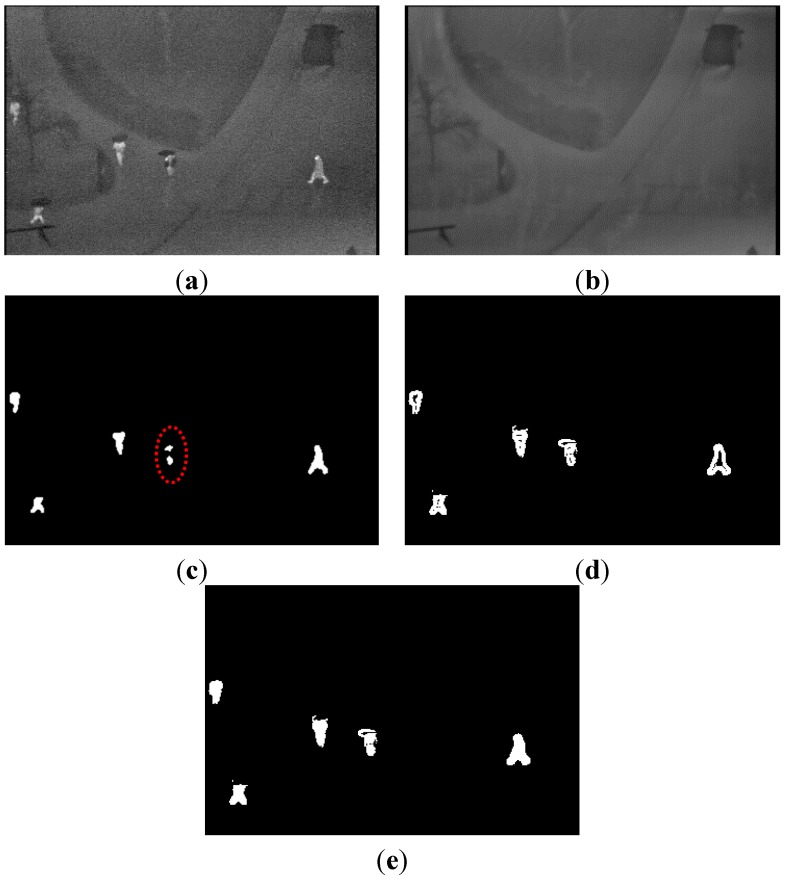
Example of the fusion of two difference images: (**a**) input image; (**b**) background image; (**c**) pixel difference image; (**d**) edge difference image; and (**e**) fusion of the pixel and edge difference images.

As indicated in Figure 6c, the difference image is created by the pixel difference between the input and background image. We can determine the rough regions of the human candidates using these procedures. However, using the pixel difference only is ineffective for detecting the shape of the objects. If only the pixel difference is used, it is difficult to detect regions of human areas whose intensities are similar to those of the background image. Furthermore, some regions of human areas can be removed or separated as small parts (as indicated in the two small areas within the red-dotted circle in the middle portion of Figure 6c) that may be removed by the subsequent filtering procedures, even though these regions contain humans.

To overcome these problems, we also create an edge difference image with edges between the background and input image. Using a standard Sobel mask of 3 × 3 pixels, the edge of the background and input image can be extracted. Based on the same methods of Equations (4) and (5), an edge image is obtained by background subtraction with the input and background edge images considering the brightness of the background image (Figure 6d).

The pixel and edge (binary) difference images are then combined (Figure 6e) using pixel-wise conjunction. This means that if either pixel of the pixel or edge difference image represents a human area, it is determined to be a human region.

### 2.4. Human Detection

With the combined image of pixel and edge differences (as explained in Section 2.3 and Figure 6e), human detection is performed as shown in Figure 1. Many noises of a small size exist in the combined image. In order to eliminate noises and figure out the candidate region of the human, component labeling, morphological operation (erosion and dilation) and size filtering are applied to the image. Then, we can get the regions of human candidates. However, it is often the case that more than two people can be detected as one candidate region. Therefore, the proposed method determines whether the candidate region is separated or not based on the histogram information of the region (see the details in Section 2.4.1).

#### 2.4.1. Division of Candidate Region Based on Histogram Information

**Figure 7:**
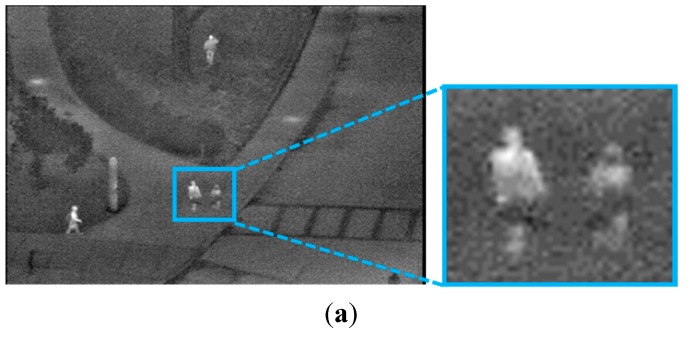

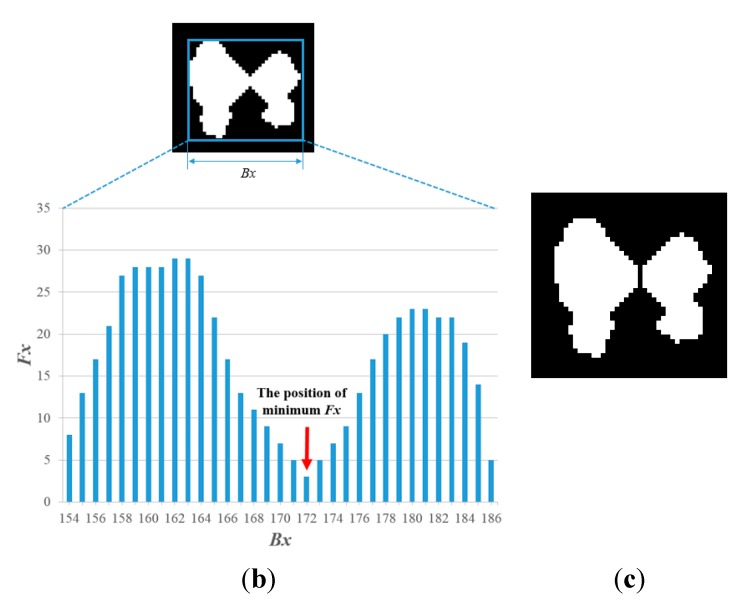
Division of the candidate region within an input image based on the horizontal histogram: (**a**) input image; (**b**) detected candidate region and its horizontal histogram; and (**c**) the division result of the candidate region.

Horizontal and vertical histograms of each candidate region are obtained to determine whether the candidate region should be separated. In Figure 7, we present an example where a candidate region is divided into two parts using the horizontal histogram based on the size, the ratio of height to width and the intensity of the background. In detail, if the size of a detected region is greater than a threshold or the ratio of the height to width is not satisfied with the condition, the region is separated into two parts based on the histogram information. This procedure is executed only if the brightness of the background is less than the threshold. This is because, in this case, the pixel and edge differences between the human area and background image become large, and consequently, the credibility of the detected candidate region is high. The horizontal histogram is obtained by Equation (6):

(6) $$Fx=\overset{I_{y}}{\underset{y=0}{\sum }}P(I(x,y))$$

where *I*(*x*, *y*) is the pixel intensity at a location (*x*, *y*) within the candidate region and *P*(·) is equal to one if *I*(*x*, *y*) is true, otherwise zero. *I_y_* is the height of the candidate region. In Figure 7b, *Bx* is the horizontal index of the candidate region within the image. As indicated in Figure 7b, if the minimum value of *Fx*, which represents the position of separation, is lower than the threshold, the candidate region is separated into two parts based on the position, as illustrated in Figure 7c.

**Figure 8:**
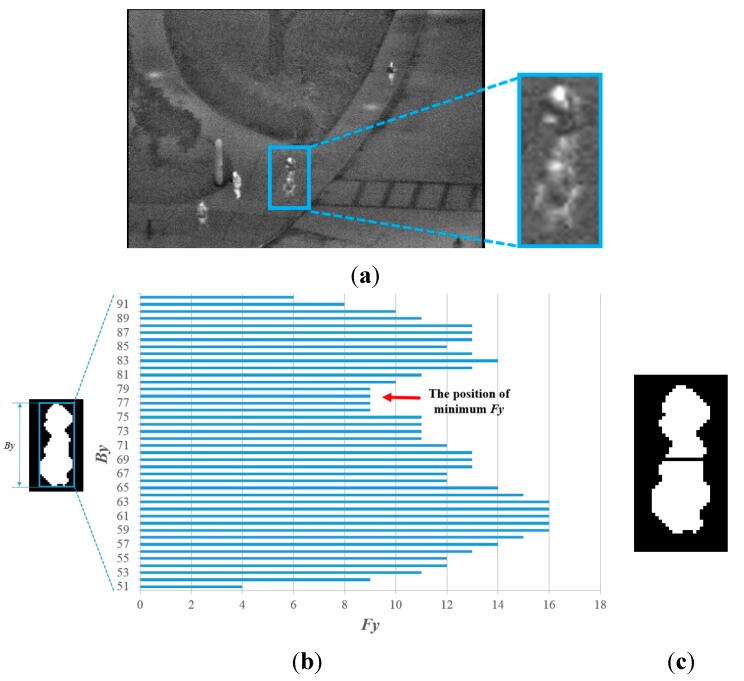
Division of the candidate region within an input image based on the vertical histogram: (**a**) input image; (**b**) detected candidate region and its vertical histogram; and (**c**) the division result of the candidate region.

Then, the proposed method determines whether the candidate region should be separated based on the vertical histogram information of the region. In Figure 8, a candidate region is divided into two parts by the vertical histogram based on the size, the ratio of the height to width and the intensity of the background. In detail, if either the size or the ratio of the height to width of the detected box is larger than the threshold considering the prior knowledge of the image area occupied by the human, the detected box is separated into two parts based on the histogram information. This procedure is executed only if the brightness of the background is less than the threshold. This is because, in this case, the pixel and edge differences between the human area and background image become large, and consequently, the credibility of the detected candidate region is high. The vertical histogram is obtained by Equation (7):

(7) $$Fy=\overset{I_{x}}{\underset{x=0}{\sum }}P(I\left(x,y\right))$$

where *I*(*x*, *y*) is the pixel intensity at location (*x*, *y*) within the candidate region and *P*(·) is equal to one if *I*(*x*, *y*) is true, otherwise zero. *I_x_* is the width of the candidate region. In Figure 8b, *By* is the vertical index of the candidate region within the image. As indicated in Figure 8b, if the minimum value of *Fy*, which represents the position of separation, is lower than the threshold, the candidate region is separated into two parts based on the position, as illustrated in Figure 8c.

#### 2.4.2. Division of the Candidate Region Based on Camera Viewing Direction with Perspective Projection

**Figure 9:**
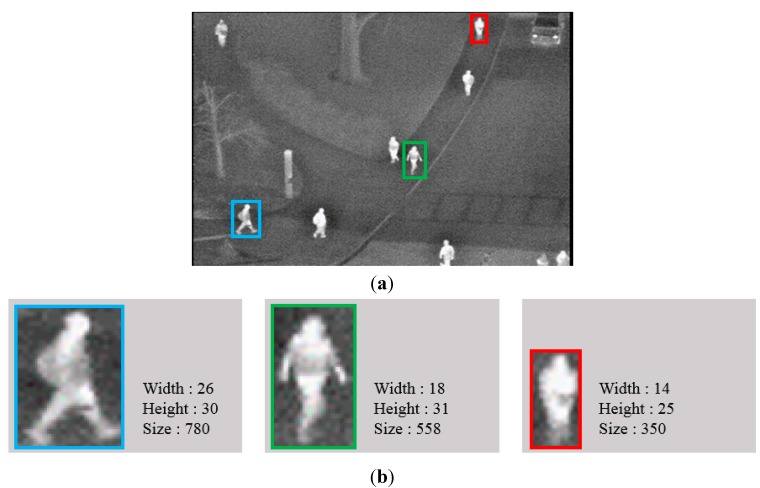
Example of different sizes of human areas resulting from camera viewing direction and perspective projection: (**a**) input image, including three detected areas of humans; and (**b**) information of the width, height and size of the three detected human areas, respectively.

To separate the candidate regions more accurately, additional procedures are performed. In Figure 9, the sizes of the candidates are considerably different from each other. This is caused by the camera viewing direction and perspective projection. Because the position of the thermal camera is near the bottom of the image and the camera captures the scene in a slanted direction, the Z distance between the camera and object in the bottom area of the image is closer than that in the upper area. Therefore, if the position of an object is located in the upper area of the image, the size of the object inevitably becomes smaller, owing to the greater Z distance based on the principle of perspective projection. Therefore, the proposed system determines whether the detected candidate is separated. In detail, a larger candidate region is allowed when it is detected in the bottom area of the image, whereas a smaller candidate region is permitted if it is detected in the upper area of the image. Candidates whose size exceeds a threshold are separated into multiple parts.

In our research, we do not actually perform the method of camera perspective projection. However, we consider only the concept of camera perspective projection (the size of the object in the camera image becomes larger with a smaller Z distance, whereas it becomes smaller with a larger Z distance) for determining whether the detected candidate should be separated or not. Therefore, actual camera calibration is not performed in our method.

**Figure 10:**
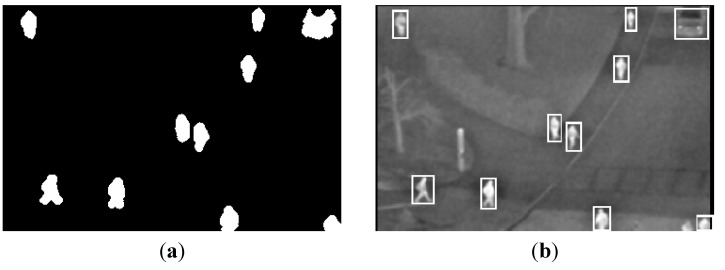

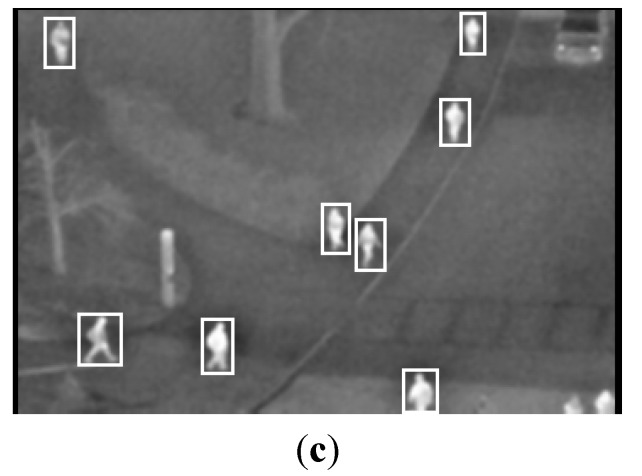
Result of obtaining the final region of the human area: (**a**) result after the process based on the separation of histogram information; (**b**) result after the process based on camera viewing direction and perspective projection; and (**c**) result of the final detected region of the human area.

Then, the correct areas of the human are detected by using size filtering based on the size and the ratio of the height to width of the region. From that, we can obtain the final human areas, excluding other objects, such as vehicles, as shown in Figure 10c.

## 3. Experimental Results

### 3.1. Dataset Description

In this research, we used two thermal databases for the experiments. As explained in Section 1, we call the image acquired in the sub-bands of MWIR and LWIR the thermal image [6] in this paper. The first database was object tracking and classification beyond visible spectrum (OTCBVS) benchmark dataset [31]. This has been widely used as an open database for the performance evaluation of object detection in thermal imaging. It contains ten categorized sequences of thermal images obtained at different times and in different weather conditions. The dataset covers various environmental conditions, such as morning, afternoon and rainy and sunny days. Each sequence contains from 18 to 73 frames, captured within one minute, such that the environmental factors, such as precipitation and temperature, remain unchanged [27]. There are 284 images. They are from 30-Hz video from an IR camera. The size of each image is 360 × 240 pixels. They were captured from the same location. Motionless humans are presented in Sequence 8. We tested all of the database images in the ten sequences.

To validate the applicability of the proposed method irrespective of the kind of database, we also created a second database with our thermal camera. For convenience, we call this “our database”. This dataset has seven categorized sequences of thermal images that were captured with different behaviors, such as walking, running, standing and sitting. Each sequence contains from 64 to 144 frames. The total number of images is 768. They were captured using an ICI 7320 thermal camera [32]. The size of each image is 320 × 240 pixels of 14 bits. Humans in the images are approximately 20 to 68 pixels in width and 34 to 103 pixels in height. Motionless humans are presented in Sequences 4, 5, 6 and 7.

We present the results of generating the background image in Section 3.2 and detection results with comparisons with other methods in Section 3.3.

### 3.2. Results of Generating Background

**Figure 11:**
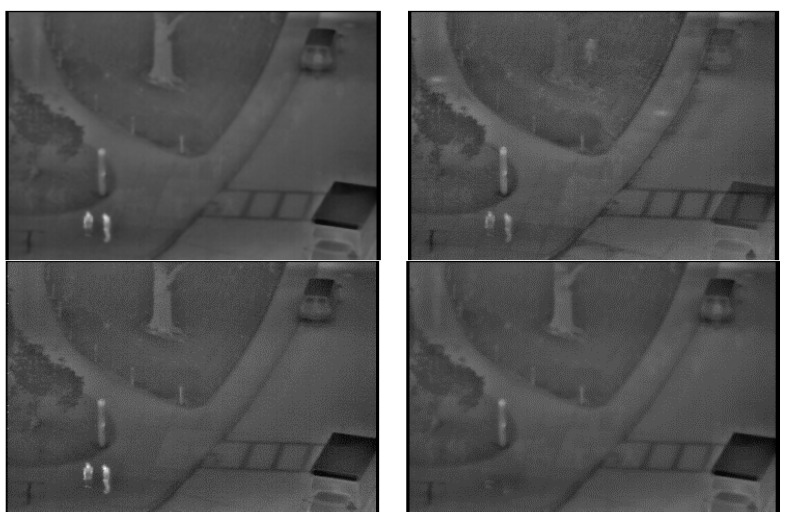
Comparisons of generated background images with the OTCBVS benchmark dataset. The left-upper [28], right-upper [26], left-lower [24,25,33], and right-lower figures are generated by previous methods and the proposed one, respectively.

To demonstrate the performance of the proposed method in generating a background, the background image from the proposed method is compared with those of other methods, as presented in Figure 11. In the previous method [28], they obtained a background image using a simple averaging operation. In [26], they obtained a background image by averaging the frames in two different sequences.

Because there exist motionless people in all of the images of Figure 11, the human areas are not completely removed from background images by the previous methods [26,28], as illustrated in the left and middle images of Figure 11, respectively. The brightness of the entire image is also changed by averaging the frames in two different sequences in [26]. This brightness change of some parts, such as trees and vehicles, causes the incorrect detection of human areas in an input image by background subtraction, because there is a difference between the brightness of the parts of background and the input images. However, all of these human areas are removed using the proposed method while maintaining the brightness of the entire image, including trees and vehicles, as displayed in the right image of Figure 11. From that, we can confirm that the correct background can be obtained using the proposed method.

Most previous research used the simple temporal averaging [26,28,34,35,36] and temporal median filtering [37] for background generation, which cannot solve the problems of motionless humans. We additionally compared other methods (not using simple temporal averaging and temporal median filtering, however; explicitly trying to solve the problems of motionless humans) [24,25,33] to our method for background generation. In the research by [24,25,33], they generated the statistical background model by calculating weighted means and variances of the sampled values. As shown in Figure 11, the human areas are not completely removed from background images by the previous methods [24,25,33], whereas all of these human areas are removed using the proposed method, which shows that our method of background generation outperforms the previous method [24,25,33].

**Figure 12:**
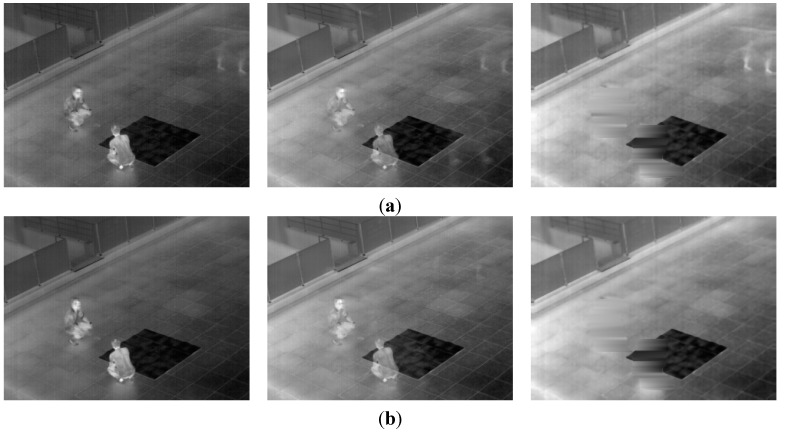
Comparisons of generated background images with our database (second database). The left, middle and right figures of (**a**,**b**) are by simple temporal averaging operation [28], averaging the frames in two difference sequences [26] and the proposed method, respectively: (**a**) with Sequence 4 of [28] (left figure) and the proposed method (right figure) and with Sequences 4 and 1 of [26] (middle figure); (**b**) with Sequence 5 of [28] (left figure) and the proposed method (right figure) and with Sequences 5 and 2 of [26] (middle figure).

In Figure 12, the background images from our database (second database) generated by the proposed method in various environmental conditions are presented. The human areas are not completely removed from the background images by the previous methods [26,28], as indicated in the left and middle images of Figure 12, respectively. Comparing the images from [26,28], the distinctiveness of the human areas is reduced significantly more by [26,28]. The brightness of the entire image is changed by averaging the frames in the two different sequences in [26]. This brightness change of the entire image causes the incorrect detection of human areas in the input image by background subtraction, because there is a difference between the brightness of the background and the input images. However, human areas are not contained in the background images from the proposed method, and we can confirm that a correct background can be obtained using the proposed method.

### 3.3. Detection Results

**Figure 13:**
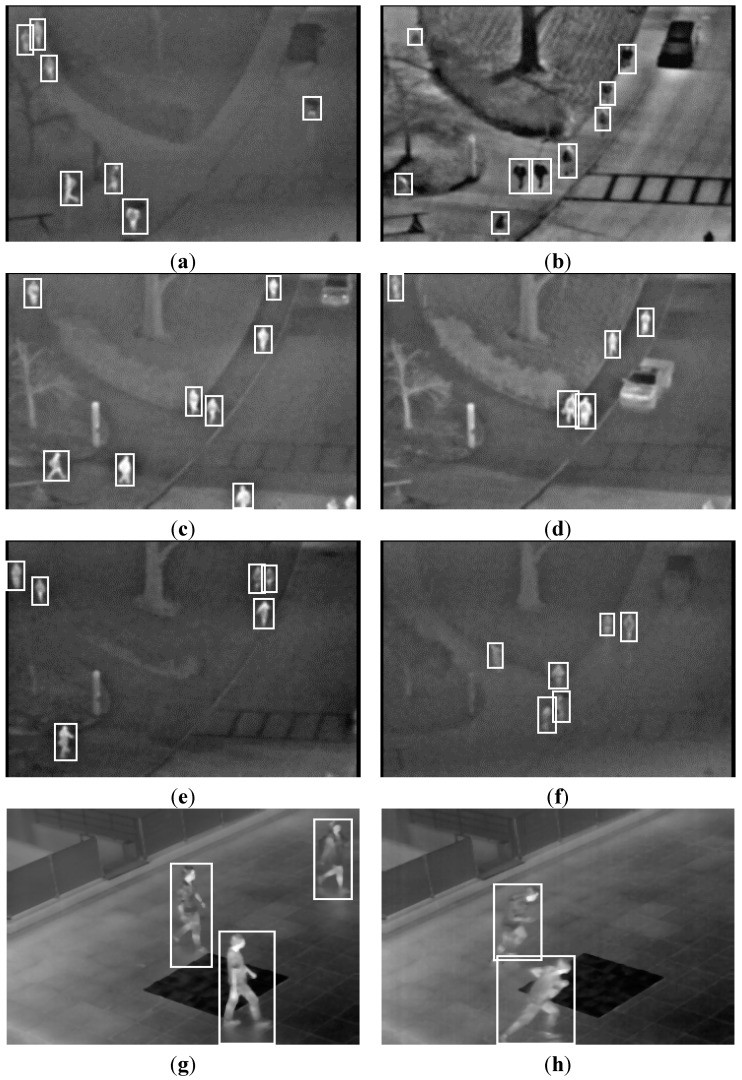

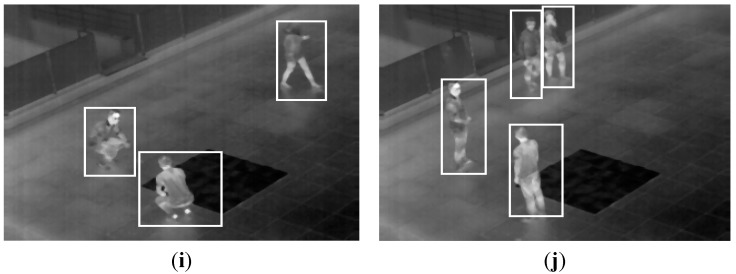
Detection results with the OTCBVS benchmark dataset (**a**–**f**) and our database (**g**–**j**). Results of images in: (**a**) Sequence 1; (**b**) Sequence 3; (**c**) Sequence 4; (**d**) Sequence 5; (**e**) Sequence 6; (**f**) Sequence 7; (**g**) Sequence 2; (**h**) Sequence 3; (**i**) Sequence 4; and (**j**) Sequence 6.

In this subsection, we present the detection results of the next experiment using the proposed method. In Figure 13, the detection results of various frames are presented. The images were captured at different times and in different weather conditions. Each detected region of a human is surrounded by a white box. In all cases, the human areas were detected successfully, in spite of the overlapping of humans (Figure 13a,d–f), darker human areas than the background (Figure 13b), vehicles, which have a similar intensity as humans (Figure 13c,d), a human using an umbrella (Figure 13a) and various kinds of human behavior, such as walking, running, sitting and standing (Figure 13g–j).

**Figure 14:**
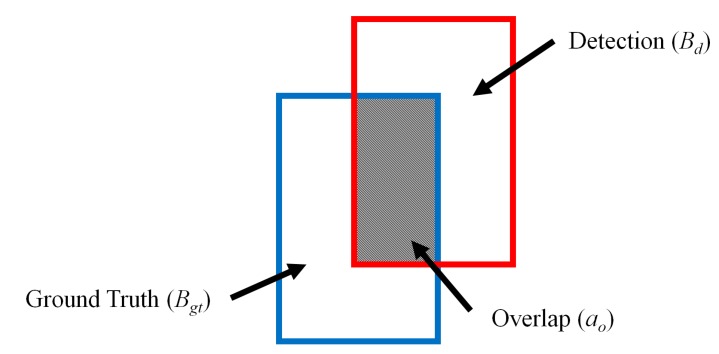
Overlapping area of ground truth and detected boxes.

In the next experiments, we quantitatively compared the detection accuracies using the proposed method to those of other methods. For this, we manually depicted bounding boxes on the human areas in the images as ground truth regions. The detection results were evaluated using the Pascal criteria [38] to judge true/false positives by measuring the overlap of the bounding box and a ground truth box. If the area of overlap $a_{o}\;$ between the detected bounding box *B_d_* and ground truth box *B_gt_* of Figure 14 exceeded 0.5 (50%) using Equation (8) [19,38,39], we counted the result as a correct detection [39].

(8) $$a_{o}=\;\frac{area(B_{d}\text{∩}B_{gt})}{area(B_{d}\text{∪}B_{gt})}$$

where *Bd* ∩ *Bgt* denotes the intersection of the detected and ground truth bounding boxes. *Bd* ∪ *B_gt_* is their union [39].

Based on Equation (8), we can count the number of true positive (TP) and false positive (FP) detection cases. Positive and negative samples mean the human area and background area, respectively. That is, the TP case is that the human area is correctly detected as a human, whereas the FP case is that the background area is incorrectly detected as a human. Based on this, we measured the accuracies of the human detection in terms of positive predictive value (PPV) (precision) and sensitivity (recall), as indicated in Equations (9) and (10) [15,40]. In these equations, #TP, #FP and #human areas in all of the images represent the number of TP cases, FP cases and human areas in all of the images, respectively. As indicated in Equations (9) and (10), a higher value of PPV and sensitivity means a higher accuracy of human detection.

(9) $$\text{PPV}=\;\frac{\#\text{TP}}{\#\text{TP}+\#\text{FP}}$$

(10) $$\text{Sensitivity}=\;\frac{\#\text{TP}}{\#\text{human areas in all the images}}$$

As indicated in Table 2, the detection accuracy of the proposed method with the OTCBVS benchmark dataset is compared with other methods [15,22,26]. Experimental results confirm that the proposed method outperformed the other methods [15,22,26] in terms of both PPV and sensitivity.

**Table 2:** Comparison of the detection results for the proposed method and other methods with the OTCBVS benchmark dataset. PPV, positive predictive value.

Sequence No.		1	2	3	4	5	6	7	8	9	10	Total
#Frames		31	28	23	18	23	18	22	24	73	24	284
#People		91	100	101	109	101	97	94	99	95	97	984
#TP	[15]	78	95	70	109	91	88	64	82	91	77	845
	[22]	88	94	101	107	90	93	92	75	95	95	930
	[26]	91	99	100	109	101	97	94	99	95	94	979
	Proposed method	91	100	99	109	101	95	94	99	95	97	980
#FP	[15]	2	3	13	10	6	2	2	0	9	0	41
	[22]	0	0	1	1	0	0	0	1	0	3	6
	[26]	0	0	2	0	0	0	0	1	0	3	6
	Proposed method	0	0	1	3	0	0	0	0	0	0	4
PPV	[15]	0.98	0.97	0.84	0.92	0.94	0.98	0.94	1	0.91	1	0.95
	[22]	1	1	0.99	0.99	1	1	1	0.99	1	0.97	0.9936
	[26]	1	1	0.98	1	1	1	1	0.99	1	0.97	0.9939
	Proposed method	1	1	0.99	0.97	1	1	1	1	1	1	0.9959
Sensitivity	[15]	0.86	0.95	0.69	1	0.83	0.91	0.68	0.83	0.96	0.79	0.86
	[22]	0.97	0.94	1	0.98	0.89	0.96	0.98	0.76	1	0.98	0.9459
	[26]	1	0.99	0.99	1	1	1	1	1	1	0.97	0.9949
	Proposed method	1	1	0.98	1	1	0.98	1	1	1	1	0.9959

In Table 3, the detection accuracy of the proposed method with our database (second database) is presented. In Table 3, the PPV and sensitivity are 98.05% and 97.35%, respectively. From Table 2 and Table 3, we can conclude that the proposed method can be applied to thermal images irrespective of the kind of database.

**Table 3:** Comparison of the detection results for the proposed method and other methods with “our database” (second database).

Sequence No.		1	2	3	4	5	6	7	Total
#Frames		137	144	64	85	127	127	84	768
#People		203	327	116	238	292	467	168	1,811
#TP	[22]	174	285	105	219	289	350	167	1,589
	[26]	203	319	98	238	292	412	168	1,730
	Proposed method	203	314	114	235	292	437	168	1,763
#FP	[22]	47	21	21	1	3	20	2	115
	[26]	1	17	0	6	0	16	52	92
	Proposed method	0	13	5	6	0	11	0	35
PPV	[22]	0.7873	0.9314	0.8333	0.9955	0.9897	0.9459	0.9882	0.9325
	[26]	0.9951	0.9494	1	0.9754	1	0.9626	0.7636	0.9495
	Proposed method	1	0.9602	0.9580	0.9751	1	0.9754	1	0.9805
Sensitivity	[22]	0.8571	0.8716	0.9052	0.9202	0.9897	0.7495	0.994	0.8774
	[26]	1	0.9755	0.8448	1	1	0.8822	1	0.9553
	Proposed method	1	0.9602	0.9828	0.9874	1	0.9358	1	0.9735

**Figure 15:**
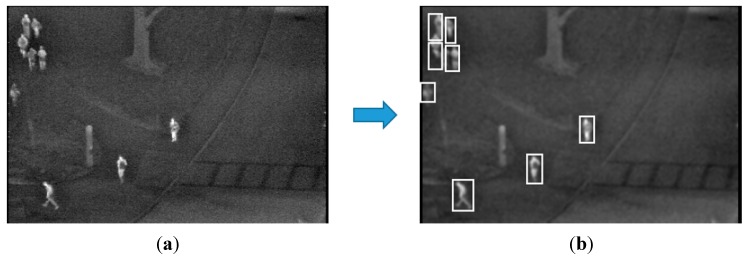
Detection error cases with the OTCBVS benchmark dataset: (**a**) original image; (**b**) result of the proposed method.

In Figure 15, we show the detection error cases by the proposed method with the open database. There are five people in the upper-left area of an image. However, four people are detected by the proposed algorithm. This is because of erroneous separation caused by the close positions of five people with occlusion.

**Figure 16:**
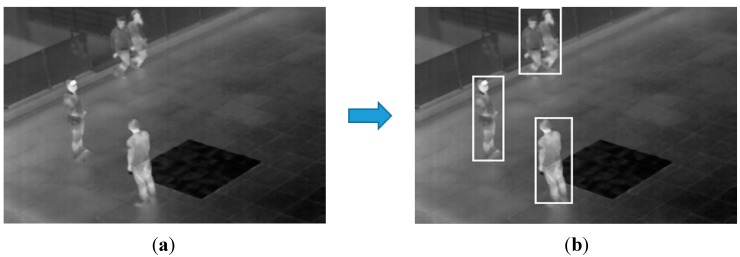
Detection error cases with our database: (**a**) original image; (**b**) result of the proposed method.

In Figure 16, we show the detection error cases by the proposed method with our database. In Figure 16, one candidate region including two people is incorrectly detected in the upper area of an image. This is because the two people are overlapped. As shown in Figure 15 and Figure 16, most of the errors are caused by the occlusion of people, and we would research the method for solving this problem based on more accurate segmentation of human areas as future work.

## 4. Conclusions

In this study, we presented a new approach to detect humans in thermal images based on the generation of a background image in various environmental conditions. A correct background image was generated by image averaging and detection and erasing methods of human areas. A difference image was obtained by combining pixel and edge difference images. Human candidate regions, detected in the difference image, were divided and redefined based on histogram information, perspective projection and intensity of the background image. Based on the redefined candidate region, correct human areas were detected. The optimal thresholds for generating difference images and defining the candidate region were adaptively determined based on the brightness of the generated background. The experimental results confirmed that the detection accuracies of the proposed method were higher than other methods.

In future work, we plan to apply the proposed method to images captured by a visible light camera. Additionally, we will research the method for solving the problem of the inaccurate detection of human areas caused by occlusion. Furthermore, we will expand the proposed research into human activity recognition.

## References

[1] Arandjelović O. Contextually Learnt Detection of Unusual Motion-Based Behaviour in Crowded Public Spaces. Proceedings of the 26th Annual International Symposium on Computer and Information Science.

[2] Martin R., Arandjelović O. (2010). Multiple-Object Tracking in Cluttered and Crowded Public Spaces. Lect. Notes Comput. Sci..

[3] Khatoon R., Saqlain S.M., Bibi S. A Robust and Enhanced Approach for Human Detection in Crowd. Proceedings of the International Multitopic Conference.

[4] Rajaei A., Shayegh H., Charkari N.M. Human Detection in Semi-Dense Scenes Using HOG descriptor and Mixture of SVMs. Proceedings of the International Conference on Computer and Knowledge Engineering.

[5] Mahapatra A., Mishra T.K., Sa P.K., Majhi B. Background Subtraction and Human Detection in Outdoor Videos Using Fuzzy Logic. Proceedings of the IEEE International Conference on Fuzzy Systems.

[6] Ghiass R.S., Arandjelović O., Bendada H., Maldague X. Infrared Face Recognition: A Literature Review. Proceedings of the International Joint Conference on Neural Networks.

[7] Bertozzi M., Broggi A., Rose M.D., Felisa M., Rakotomamonjy A., Suard F. A Pedestrian De-Tector Using Histograms of Oriented Gradients and a Support Vector Machine Classifier. Proceedings of the IEEE Conference on Intelligent Transportation Systems.

[8] Li Z., Zhang J., Wu Q., Geers G. Feature Enhancement Using Gradient Salience on Thermal Image. Proceedings of the International Conference on Digital Image Computing: Techniques and Applications.

[9] Chang S.L., Yang F.T., Wu W.P., Cho Y.A., Chen S.W. Nighttime Pedestrian Detection Using Thermal Imaging Based on HOG Feature. Proceedings of the International Conference on System Science and Engineering.

[10] Bertozzi M., Broggi A., Caraffi C., Rose M.D., Felisa M., Vezzoni G. (2007). Pedestrian Detection by Means of Far-Infrared Stereo Vision. Comput. Vis. Image Underst..

[11] St-Laurent L., Prévost D., Maldague X. Thermal Imaging for Enhanced Foreground-Background Segmentation. Proceedings of the International Conference on Quantitative InfraRed Thermography.

[12] Lin C.F., Lin S.F., Hwang C.H., Chen Y.C. Real-Time Pedestrian Detection System with Novel Thermal Features at Night. Proceedings of the IEEE International Instrumentation and Measurement Technology Conference.

[13] Zhao J., Cheung S.C.S. Human Segmentation by Fusing Visible-light and Thermal Imaginary. Proceedings of the IEEE International Conference on Computer Vision Workshops.

[14] Chen Y., Han C. Night-Time Pedestrian Detection by Visual-Infrared Video Fusion. Proceedings of the World Congress on Intelligent Control and Automation.

[15] Li W., Zheng D., Zhao T., Yang M. An Effective Approach to Pedestrian Detection in Thermal Imagery. Proceedings of the International Conference on Natural Computation.

[16] Neagoe V.E., Ciotec A.D., Barar A.P. A Concurrent Neural Network Approach to Pedestrian Detection in Thermal Imagery. Proceedings of the International Conference on Communications.

[17] Zhang L., Wu B., Nevatia R. Pedestrian Detection in Infrared Images Based on Local Shape Features. Proceedings of the IEEE Conference on Computer Vision and Pattern Recognition.

[18] Olmeda D., Armingol J.M., Escalera A.D.L. Discrete Features for Rapid Pedestrian Detection in Infrared Images. Proceedings of the IEEE/RSJ International Conference on Intelligent Robots and Systems.

[19] Wang W., Zhang J., Shen C. Improved Human Detection and Classification in Thermal Images. Proceedings of the IEEE International Conference on Image Processing.

[20] Wang W., Wang Y., Chen F., Sowmya A. A Weakly Supervised Approach for Object Detection Based on Soft-Label Boosting. Proceedings of the IEEE Workshop on Applications of Computer Vision.

[21] Davis J.W., Sharma V. Robust Detection of People in Thermal Imagery. Proceedings of the International Conference on Pattern Recognition.

[22] Davis J.W., Keck M.A. A Two-Stage Template Approach to Person Detection in Thermal Imagery. Proceedings of the IEEE Workshop on Applications of Computer Vision.

[23] Latecki L.J., Miezianko R., Pokrajac D. Tracking Motion Objects in Infrared Videos. Proceedings of the IEEE International Conference on Advanced Video and Signal Based Surveillance.

[24] Davis J.W., Sharma V. (2007). Background-Subtraction Using Contour-Based Fusion of Thermal and Visible Imagery. Comput. Vis. Image Underst..

[25] Davis J.W., Sharma V. Fusion-Based Background-Subtraction Using Contour Saliency. Proceedings of the IEEE Computer Society Conference on Computer Vision and Pattern Recognition—Workshops.

[26] Dai C., Zheng Y., Li X. Layered Representation for Pedestrian Detection and Tracking in Infrared Imagery. Proceedings of the IEEE Computer Society Conference on Computer Vision and Pattern Recognition—Workshops.

[27] Dai C., Zheng Y., Li X. (2007). Pedestrian Detection and Tracking in Infrared Imagery Using Shape and Appearance. Comput. Vis. Image Underst..

[28] Calafut M. Multiple-Object Tracking in the Infrared, Final Project (EE368) of Stanford University.

[29] Li J., Gong W. (2010). Real Time Pedestrian Tracking Using Thermal Infrared Imagery. J. Comput..

[30] Niblack W. (1986). An Introduction to Digital Image Processing.

[31] OTCBVS Benchmark Dataset Collection. http://www.cse.ohio-state.edu/otcbvs-bench/.

[32] ICI 7320 Scientific Specifications. http://www.infraredcamerasinc.com/Thermal-Cameras/Fix-Mounted-Thermal-Cameras/ICI7320_S_fix-mounted_thermal_camera.html.

[33] Davis J.W., Sharma V. (2007). Background-Subtraction in Thermal Imagery Using Contour Saliency. Int. J. Comput. Vis..

[34] Dagless E.L., Ali A.T., Cruz J.B. Visual Road Traffic Monitoring and Data Collection. Proceedings of the IEEE-IEE Vehicle Navigation and Information Systems Conference.

[35] Elhabian S.Y., El-Sayed K.M., Ahmed S.H. (2008). Moving Object Detection in Spatial Domain Using Background Removal Techniques-State-of-Art. Recent Pat. Comput. Sci..

[36] Zheng Y., Fan L. Moving Object Detection Based on Running Average Background and Temporal Difference. Proceedings of the International Conference on Intelligent Systems and Knowledge Engineering.

[37] Malviya A., Bhirud S.G. (2010). Visual Infrared Video Fusion for Night Vision Using Background Estimation. J. Comput..

[38] Olmeda D., Premebida C., Nunes U., Armingol J.M., Escalera A.D.L. (2013). Pedestrian Detection in Far Infrared Images. Integr. Comput. Aided Eng..

[39] Everingham M., Gool L.V., Williams C.K. I., Winn J., Zisserman A. (2010). The Pascal Visual Object Classes (VOC) Challenge. Int. J. Comput. Vis..

[40] Sensitivity and Specificity. http://en.wikipedia.org/wiki/Sensitivity_and_specificity.

